# The Application of Metagenomics to Study Microbial Communities and Develop Desirable Traits in Fermented Foods

**DOI:** 10.3390/foods11203297

**Published:** 2022-10-21

**Authors:** Meghana Srinivas, Orla O’Sullivan, Paul D. Cotter, Douwe van Sinderen, John G. Kenny

**Affiliations:** 1 Food Biosciences Department, Teagasc Food Research Centre, Moorepark, P61 C996 Cork, Ireland; 2 APC Microbiome Ireland, University College Cork, T12 CY82 Cork, Ireland; 3 School of Microbiology, University College Cork, T12 CY82 Cork, Ireland; 4 VistaMilk SFI Research Centre, Fermoy, P61 C996 Cork, Ireland

**Keywords:** fermented foods, omics, metagenomics, food quality, traceability, microbial community

## Abstract

The microbial communities present within fermented foods are diverse and dynamic, producing a variety of metabolites responsible for the fermentation processes, imparting characteristic organoleptic qualities and health-promoting traits, and maintaining microbiological safety of fermented foods. In this context, it is crucial to study these microbial communities to characterise fermented foods and the production processes involved. High Throughput Sequencing (HTS)-based methods such as metagenomics enable microbial community studies through amplicon and shotgun sequencing approaches. As the field constantly develops, sequencing technologies are becoming more accessible, affordable and accurate with a further shift from short read to long read sequencing being observed. Metagenomics is enjoying wide-spread application in fermented food studies and in recent years is also being employed in concert with synthetic biology techniques to help tackle problems with the large amounts of waste generated in the food sector. This review presents an introduction to current sequencing technologies and the benefits of their application in fermented foods.

## 1. Introduction

The use of fermented foods has been recorded for thousands of years and continues to be of global importance to this day. Numerous fermented foods have existed across civilizations with the techniques used being indigenous to the resources available to a region. Traditionally, fermentations were carried out as a method of preservation to improve the microbiological safety while prolonging the shelf-life of food products, or for the exclusion of pathogens, when cold storage methods were not yet invented [1]. Over the years, however, fermented foods have been exploited for their health-promoting activities, and for their appeal to the consumer and industry, leading to their large-scale production [2,3,4]. The extent to which they have been commercialised depends on the region they are from, the techniques used, the availability of starter cultures used to start the fermentation process, along with resources available to research and industrialise the process [1]. A vast number of fermentation processes have been largely amenable to industrialisation where the starter cultures and production techniques have been well-characterised and fine-tuned over decades to produce consistent and high-quality products as in the case of the dairy, bread, meat and brewing industries. Others have remained very traditional, with recipes passed down from one generation to another in a household, or for small-scale production in local cottage industries. These methods depend largely on existing, yet undefined starter cultures that are added to start fermentations through a method known as back-slopping where an amount of a previous batch of fermented product is added to start a new fermentation [5]. However, since the starter cultures involved are still largely uncharacterised and little quality control can be performed while back-slopping, consistency and microbiological safety of the product remains to be a matter of concern, often making it inefficient for industrial fermentations [6]. Another food fermentation method known as spontaneous fermentation, uses naturally occurring microbes that are native to the raw food matrix and surrounding environment to carry out fermentations. Examples of spontaneous fermentations include the production of sour beers, some wines, and vegetable based-fermentations such as sauerkraut and kimchi [7,8,9,10].

Fermentation microbiomes are complex and dynamic with various microbes imparting characteristic flavours, odours, and texture throughout the fermentation process and into the finished product [11,12,13,14]. Profiling and characterisation of starter cultures and autochthonous fermentation microbes provides clarity in understanding underlying fermentation principles and allows optimisation of fermentation processes to improve product organoleptic and microbial safety qualities, and to ensure product consistency. This is where metagenomics plays a major role by allowing microbial characterisation and tracking, while providing insight into their interactions with other members of the fermented food microbial community.

The development of high-throughput sequencing (HTS) has allowed the application of metagenomics in numerous environmental and more recently fermentation microbiome studies [15]. With metagenomics, the entire DNA content of a microbial community can be studied at the same time, unlike culture-dependent methods where single colonies are isolated in order to sequence their whole genomes. Being a culture-independent technique, metagenomics is able to identify and characterise microbial species that are difficult to grow in a lab setting [16]. However, sequencing dead microbial cells confounds metagenomics data analysis. The inadequate detection of microbial populations present at low relative abundance is also problematic to the application of metagenomics to highly diverse and/or low microbial abundance samples. The potential solutions to these issues are described in the following sections.

Metagenomic sequencing can be broadly classified into two methods based on the DNA regions being sequenced, one being targeted or amplicon sequencing, also termed as metabarcoding or metataxonomics [6], where specific regions of the gDNA in a microbiome sample is targeted by PCR amplification and sequencing, and the other being untargeted or shotgun metagenomic sequencing where the entire genetic material in a microbiome sample is sequenced. The sequencing platform or method chosen depends largely on the type and number of samples, budget of the project, and computational resources available to process and analyse the sequencing data [17]. After sample collection and storage, a metagenomics experiment can broadly be broken down into four main steps; (i) extraction of microbial DNA from the sample; (ii) library preparation; (iii) DNA sequencing; (iv) bioinformatic processing and analysis of the generated sequence data [18].

## 2. Microbial DNA Extraction

The success of sequencing in terms of data quality and output is to a large degree dependent on the quality of DNA extracted from microbial communities. The DNA input requirements, such as concentration and fragment length, vary based on the sequencing method and platform used. Damaged and fragmented DNA can often lead to problems with library preparation, causing inefficient sequencing. DNA isolation can be especially difficult when collecting samples from equipment and food contact surfaces in food processing facilities due to the use of sanitising agents on these surfaces that injure micro-organisms and nick the DNA of the microbes present [19,20]. Unfermented and fermented foods are often rich in lipids and proteins, which can interfere with microbial DNA extractions, and must be removed prior to DNA extraction [21,22]. Pre-DNA extraction processing methods for traditional fermented foods have also been developed for highly viscous and sticky fermented foods rich in microbial polysaccharides and that are otherwise difficult to process [23,24,25]. After pre-processing samples, when required, metagenomic DNA extraction is performed. A number of non-commercially and commercially available DNA extraction kits exist, with each approach having its own advantages and disadvantages depending on the sample type and sequencing method employed [26,27]. Commercial DNA extraction kits can be expensive and may not be applicable to very traditional fermentation setups. However, they have the advantage of being standardised approaches [23].

Long read sequencing can be particularly sensitive to the quality of input DNA as highly fragmented DNA can only produce short reads, thereby failing to realise the advantages of the sequencing platform. Various commercially available DNA extraction kits are recommended by PacBio for long read sequencing [28], and have been used for long read 16S rRNA metabarcoding [29,30,31]. These methods involve mechanical bead beating steps that some consider detrimental to the success of long read sequencing while others consider this a requirement to provide good representations of highly diverse microbial communities [32,33,34,35]. However, mechanical steps involved in extraction procedures can be harsh on the DNA leading to fragmented DNA templates, which may underutilize the potential of long read sequencing platforms [36]. DNA extraction methods specifically suited to long read sequencing, called High Molecular Weight (HMW) DNA extractions, have therefore been developed to avoid bead-beating steps. MetaPolyzyme (a commercial product sold by Sigma Aldrich, Burghausen, Germany), is one such example, where an enzyme cocktail is used instead of mechanical steps to lyse microbial cells [37]. Kits for HMW DNA extractions for metagenomics are also commercially available with a few examples being the DNA extraction kits by Circulomics and “Fire Monkey” by RevoluGen.

## 3. Host Depletion

When assessing the quality of DNA extracted for metagenomic sequencing, contamination from non-microbial or host DNA, usually animal or human, should also be considered. More than 90% of the DNA fragments from samples such as blood, saliva and milk can come from the host genomes [38,39]. In shotgun metagenomics, since all the genetic material including host DNA is sequenced, a large amount of sequencing output is wasted on such contaminating host DNA [40,41]. This can lead to a high number of microbial species being unclassified, incorrectly classified and/or under-represented, thus causing serious inaccuracies in sample microbial community profiling [42,43]. This is especially problematic when applying shotgun sequencing to low microbial abundance samples such as saliva, skin and milk [44]. Therefore, host DNA depletion is often advantageous when preparing gDNA for shotgun sequencing. Host depletion in amplicon-based approaches is not required as the PCR step is selective and amplifies only target microbial DNA regions [41].

Host DNA depletion can be performed in two ways depending on whether they are carried out pre- or post-extraction. Pre-extraction methods use chaotropic agents to lyse mammalian host cells while allowing microbial cells to remain intact. The released host DNA is then degraded by nucleases such as DNase I or Benzonase. The latter is increasingly employed due to its wide range of operating conditions compared to DNase I. Once host DNA is degraded, microbial gDNA extraction is performed [41]. Commercial kits such as MolYsis (Molzym, Bremen, Germany) use this pre-extraction method with a proprietary DNase called MolDNase B, while the QIAmp DNA Microbiome kit (Qiagen, Hilden, Germany) performs host depletion using Benzonase [45]. In food metagenomics, the MolYsis kits were observed to be well suited for milk metagenome studies [21,44]. The Host ZERO microbial DNA kit (Zymo, Irvine, CA, USA) also uses the pre-extraction method with bead beating using two different bead sizes for host depletion [45]. Propidium Monoazide (PMA)-based methods for host depletion are also available that are performed prior to extraction [46]. The drawbacks observed for pre-extraction methods are: (i) the likely destruction and consequent under-representation of sensitive microbes such as *Mycoplasma* spp. and parasites during selective lysis, and (ii) for PMA-based methods, biasing towards Gram-positive bacteria due their increased susceptibility to PMA treatment compared to Gram-negative bacteria [34,41,47,48]. The second approach to host DNA depletion takes place post-DNA extraction, and uses differences in methylation characteristics between microbial and host or eukaryotic genomes. The NEBNext Microbiome DNA Enrichment kit (NEB, Northborough, MA, USA) uses magnetic beads to selectively bind and remove CpG methylated host DNA. However, with this post-extraction method, problems have been identified for AT rich genomes and differentiating between eukaryotic microbial and host DNA such as fungi, algae and protozoa that have similar methylation patterns [41]. In general, the method used for host depletion can vary between sample types with each having their own drawbacks and so should be decided accordingly [49]. Both pre- and post-extraction methods involve a number of washing and spinning steps that can reduce microbial (DNA) abundances in samples [45]. This is a major problem in low biomass samples, sometimes requiring PCR steps to obtain sufficient DNA concentrations for library preparation [50].

## 4. Differentiating between Live and Dead Bacteria

Differentiating between viable and non-viable microbes in a sample community can provide useful information and is performed through a process known as viability testing. In a microbial community, microbial populations can shift over time with various microbial species dominating and dying out. This shift in microbial populations can affect the type and quantity of metabolites produced which can affect neighbouring microbes and the surrounding environment [43,47,51]. While metagenomics provides information on the entire microbial community, by itself it cannot differentiate between live and dead bacteria. For a better understanding of microbial communities at particular time-points, additional methods to differentiate between live and dead bacteria are needed to be applied [52,53]. Propidium Monoazide (PMA) is the most commonly employed for viability testing. PMA is a dye that intercalates with DNA in the absence of a cell membrane. Upon exposure to visible light, PMA undergoes cleavage in its azide group with a C-H insertion reaction leading it to being covalently bound with the DNA. In this way, PMA acts only on free DNA released from dead and/or membrane-damaged microbial cells to prevent their further processing and sequencing [46]. The sequencing data obtained will therefore be representative of the viable microbial cells at a specific time-point. While PMA offers the benefits of viability testing, its activity has been assessed only on a small subset of biological matrices. A number of factors such as sample type, chemical composition, experimental conditions, duration of light exposure, and incubation time can influence PMA’s activity in degrading free DNA. Some cases have been reported where PMA partially or completely fails to remove free DNA, which can skew the results obtained, leading to under and/or mis-representations of the microbial community [54,55]. PMA penetration into dead cells also may be incomplete and may not be permitted in partially membrane-compromised bacterial cells, which can result in overestimations of live cells [56,57]. Therefore, the use of PMA in viability-based metagenomics needs to be further standardised. Live, metabolically active microbes in a sample can also be selected for and characterised using methods such as metatranscriptomics and metaproteomics where only mRNA or actively-expressed proteins, respectively, are sequenced [53,56,58]. Both metatranscriptomics and metaproteomics have been useful in understanding fermentation microbiomes and the interactions within its communities [59,60,61].

## 5. Sequencing Platforms

Sanger sequencing was among the first generation of sequencing technologies that largely contributed to the development of automated DNA sequencers [62]. Since then, major advances in sequencing technology has led to the rise of Next Generation Sequencers (NGS) that marked the start of many of the short read and metagenomic applications presently seen. Roche 454, Illumina, and Ion Torrent have been the forerunners of NGS with a vast majority of metagenomic projects employing the Illumina suite of sequencers [62,63,64,65]. Illumina platforms use sequencing by synthesis, which occurs on flow cells and uses fluorescently labelled nucleotides which are incorporated by DNA polymerases complementary to the template DNA strand. On incorporation, light of a specific wavelength is emitted and images are taken by a camera in the instrument. The images are then interpreted to DNA sequences one base at a time [65]. The high throughput, relatively low cost per base, and low error rates of 0.1–1% in Illumina sequencers is the reason behind the platform dominating the short read sequencing market [66]. As the demand for improved sequencing methods is increasing, the recent releases of Illumina aim at improving the throughput capacity and cost efficiency, while also reducing error rates. Among the latest Illumina releases is the NovaSeq 6000, which allows industrial-scale sequencing, generating up to 6 Tb and 20 billion reads with the lowest cost per base compared to earlier versions. Reduced error rates have also been recorded on NovaSeq 6000 and HighSeq X Ten, with the latter of the two sequencers being the most inexpensive human genome sequencer [67].

Over recent years there have also been ever greater developments relating to the third generation of sequencers, i.e., long read sequencers. PacBio and Oxford Nanopore Technologies (ONT) have dominated much of the long read sequencing market. The principle used in PacBio sequencing is that DNA fragments of approx. 250 to 25,000 bp are ligated with hairpin adapters forming a circular template, which when introduced to the Single Molecule Real Time (SMRT) cell, settle in the wells of the cell with one circular template taking up one well each. Within the wells, DNA polymerases add nucleotides complementary to the template DNA strand. This process can happen either multiple times in a mode known as Circular Consensus Sequencing (CCS) to generate HiFi data that is of high accuracy, or in a mode wherein longer DNA templates will be sequenced fewer times with more importance given to sequencing the entire length of the DNA fragment generating continuous long read (CLR) data [68]. A mix of the two methods, CCS and CLR, have been applied to sequence long eukaryotic genomes [69,70,71]. ONT uses protein pores, called nanopores, which are embedded into a membrane on a flow cell. During sequencing, an ion current is applied and single stranded DNA moves through the nanopores. As the DNA passes through a nanopore a characteristic disruption in ion current is identified by sensors and recorded. These recorded disruptions are then analysed to determine the corresponding nucleotide sequences. When HMW DNA extraction methods are followed, ONT platforms can even generate reads of 1 Mb in length or even longer [72].

PacBio and ONT have found application in both amplicon and shotgun metagenomics to varying extents. One of the major drawbacks in both the platforms was the historical high raw error rates of about 10–20% [73]. However, recently numerous studies have been dedicated to addressing this issue and has resulted in a number of bioinformatic tools and pipelines available for reducing and correcting error rates in long read platforms [73,74,75]. Significant improvements are also being made by PacBio and ONT with frequent releases of kit chemistries, sequencing instruments and flow cells allowing improved efficiency, accuracy, and data yield making it more amenable to wider application in metagenomic studies. The latest Sequel II and Sequel IIe platforms by PacBio along with the new 8M flow cells can provide accuracy of 99.8%, comparable to that of short read sequencing [68,72]. The recently released kit 12 chemistry and R10.4 cells by ONT supported by 1D2 technology allows consensus sequencing of complementary DNA strands and has an increased sequencing accuracy of more than 99% [76,77].

Apart from the sequencing platforms that currently dominate much of the market, newer competing platforms have recently been introduced that improve the scope of accessibility of sequencing technologies. Examples include Element Biosciences, MGI, and Omniome. All three target improvements in data accuracy and yield, alongside cost reduction, which will hopefully benefit customers/consumers due to increased competition in the short-read sequencer market.

## 6. Library Preparation and Multiplexing

Library preparation can be divided into the following steps: DNA processing to obtain PCR amplicons or fragments of desired sizes, multiplexing, and in most cases adapter ligation with the exception of amplicon sequencing on Illumina platforms.

For amplicon sequencing, amplicons are generated by targeting gDNA regions through PCR amplification. The amplicon size and PCR conditions depend on the sequencing platform and objective of the study. For shotgun sequencing, DNA fragments of desired size are obtained through a process known as fragmentation, which can be performed using sonication, acoustic cavitation, or enzymatically with DNA nucleases. DNA fragments of less than 450 bp are recommended for short read sequencing platforms, while fragment lengths up to 75 kb are often desirable for long read sequencing [78]. Often this means a need to isolate HMW DNA as described above. Sometimes it is still useful to fragment HMW to smaller fragments of ~20 kb to improve yields of sequencing, or to allow for multi-pass HiFi reads. In such cases, specific mechanical shearing devices such as the Megaruptor system are used as they improve consistency and reproducibility of the fragment lengths [79,80]. Post fragmentation, size selection for the desired fragment lengths is often performed.

Multiplexing, also called indexing or barcoding, is a method in which multiple libraries are pooled together so they can be sequenced on a single run and is used to reduce cost and save time when sequencing a large number of samples. Multiplexing uses specific and distinct nucleotide sequences, called index sequences or barcodes, which are added to the ends of amplicons or DNA fragments. After sequencing, barcoding allows assignment of the sequencing reads to the specific source sample from the pool of libraries [81].

**Figure 1:**
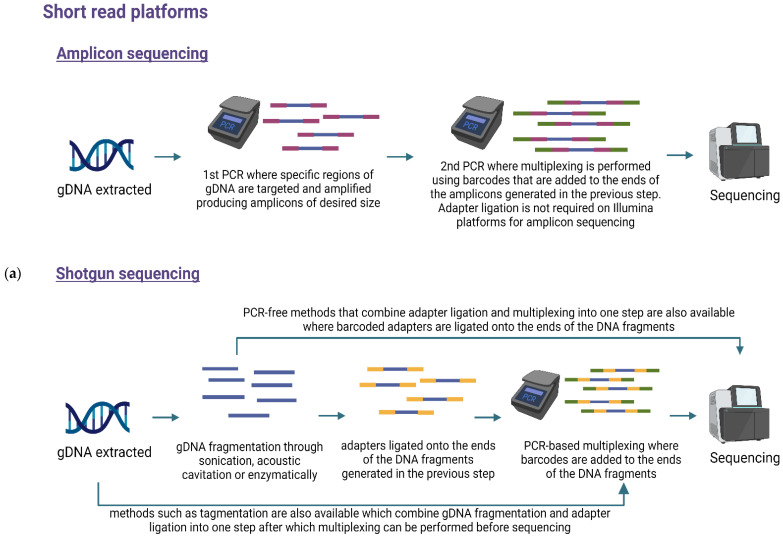

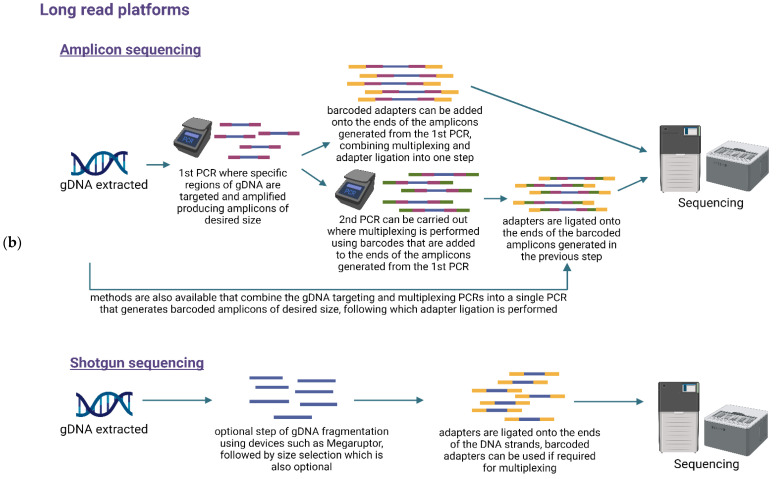
Overview of library preparation steps for amplicon and shotgun sequencing on (**a**) short read platforms such as Illumina, and (**b**) long read platforms such as PacBio and ONT [72,82]. This figure was created with BioRender.com.

Adapter ligation is the process in which platform-specific nucleotide sequences are added onto the ends of amplicons or DNA fragments, which allow the DNA regions of interest to bind or settle in the flow cells where sequencing occurs. For shotgun sequencing on Illumina, the adapters help bind the template DNA to the flow cell where sequencing cycles take place [82]. Amplicon sequencing on Illumina does not require adapter ligation because the adapter sequences can be incorporated during PCR. In PacBio, the hairpin adapters provide a circular shape to the long DNA fragments before the DNA polymerases initiate sequencing. In ONT, adapters are ligated to double stranded DNA and allows the strands to be captured by the nanopores on the flow cell. The ONT adapters also act as the starting point for a motor enzyme that runs along a DNA strand helping it pass through the nanopore [72]. The specific processes and order in which multiplexing and adapter ligation is carried out during library prep depends on the sequencing platform, kits used and the sequencing method. Figure 1 presents a general overview of their workflow.

While multiplexing is advantageous, there are a few challenges that are yet to be overcome in the technology. Misassignment of reads to indexes, and so their source libraries, is a common problem on various sequencing platforms leading to issues in downstream analysis [83]. It has been identified as a particular problem with Illumina sequencers using patterned flow cells due to the chemistries involved [84]. This problem of “index hopping” has been linked to the presence of free-floating indexing primers present in the pooled libraries introduced onto the flow cell [85,86]. Ineffective clean up and size selection steps, and improper storage of the prepared libraries leading to fragmentation of the template DNA, are sources of these free-floating indexing primers in the pooled libraries [86]. One solution to this issue is the use of unique dual indexing, where the indexing sequences added on either side of the amplicon or DNA fragment is unique to a single library. This means every library has two index sequences, one at each end of the DNA fragments that are unique to it. No index sequence will be shared between any two or more libraries of that pool [87]. However, the need for high numbers of validated indexes, and the associated costs with having so many indexes available can make unique dual indexing challenging when pooling a large number of samples. In these situations nested metabarcoding, where a combination of two indexing primer pairs are incorporated onto the ends of the template DNA through a nested PCR approach, can be used instead. This allows four distinct indexing primers to be incorporated within each library fragment to minimise the effects of index hopping [81,88]. Cross-talk between indexing primers can also occur by other means, including cross-contamination during the synthesis of primers or adapters, sample handling, the generation of chimeras during PCR steps, multiple misread of bases in the index sequences during sequencing, and carry-over of indexing primers or adapters from previous sequencing runs [83,87]. Many of these sources of error can be eliminated by following good laboratory and library prep practices [16]. However, index hopping continues to be an area of concern with newer sequencing companies such as MGI introducing methods claiming to have reduced index hopping on their platforms [89].

The reagents used for extraction and library preparation are another source of bias in metagenomic sequencing. Microorganisms have been found to grow in the buffers and reagents used in DNA extraction and library preparation, such as in the PCR reagents or water. This contaminating microbial DNA is sequenced along with the intended metagenomic samples, biasing the microbial community representations and causing inaccuracies in taxonomic classifications, microbial abundance and diversity calculations [90]. Shotgun metagenomics, especially for low biomass samples, are also very sensitive to this so-called “kitome” contamination. This makes the inclusion of experimental controls such as mock communities and negative control extractions of paramount importance to remove these sources of bias [16,91].

## 7. Sequencing Methods

### 7.1. Targeted or Amplicon-Based Sequencing

The DNA regions most often targeted in metabarcoding is the 16S rRNA gene in bacteria and the Internal Transcribed Spacer (ITS) region in fungal studies [92,93]. The 16S gene has been chosen for metabarcoding in bacterial genomes as it is largely conserved in almost all bacterial species allowing the use of universal primers, while hypervariable regions permit the identification and taxonomic classification of bacteria. The 16S rRNA gene plays a crucial role in protein synthesis initiation and mRNA translation and is present in every bacterial cell, making it a universal target [94]. Short read sequencing only allows some of the hypervariable regions (designated V1 through to V9) of the 16S rRNA gene to be sequenced. Generally amplicons of up to 450 bp to include regions such as V1–V3, or V3–V4 are targeted by PCR for sequencing. The appropriateness of the hypervariable regions depends on the nature of sample source. Debate remains in this area as hypervariable regions targeted between different studies and for specific bacterial genera differ [19,95,96,97]. Irrespective of the issue relating to the choice of hypervariable regions used, 16S rRNA sequencing has seen massive application in the metagenomics field, specifically for the V3–V4 region coupled with Illumina sequencing [98,99,100,101]. A majority of metabarcoding studies have employed the Illumina MiSeq or HiSeq 2500 platforms, the latter of which is no longer supported.

The relative ease with which bioinformatic processing and analysis of amplicon data can be performed compared to shotgun metagenomic data is another contributing factor to the widespread application of metabarcoding. The processing and analysis usually involves quality control steps of quality trimming, quality filtering and adapter removal from the reads, followed by taxonomic classification, which is usually performed using alignment methods against reference databases. For short reads, taxonomic classifications can be performed either through clustering sequences, often with 97% similarity, into Operational Taxonomic Units (OTUs), or by grouping of identical or exact matching sequences using Amplicon Sequencing Variants (ASVs). QIIME2 [102], mothur [103,104], MG-RAST [105], UPARSE [106], FROGS [107] are examples of OTU based pipelines while, Bioconductor [108], Deblur [109], and DADA2 [110] are examples of ASV-based pipelines. ASV-based methods have been found to provide better resolution than OTU-based methods [111]. A detailed discussion of 16S analysis pipelines is beyond the scope of this review, and for more information we refer to some excellent reviews [112,113].

Metabarcoding using long read sequencing has developed substantially over the recent years with improvements in base calling, reduced error rates, and fine tuning of bioinformatic pipelines [114]. Many fermented food studies have applied full length sequencing of the 16S gene (approx. 1500 bp in size) to determine microbial communities [19,29,30,31,115,116]. Compared to short read sequencing of one or two hypervariable regions, long read sequencing of the entire 16S gene does improve resolution of taxonomic assignments from genus level to species level. This avoids problems associated with the choice of which hypervariable regions to target, but strain level resolution still cannot be obtained. As a solution to this, attempts have been made to use long read amplicon sequencing to target the entire RRN operon (approx. 4300 bp in size) consisting of the 16S rRNA gene, ITS region and 23S rRNA gene [117]. Targeting the combined 16S-ITS-23S regions instead of individual rRNA locus-derived fragments as commonly done in short read metabarcoding, can provide information on 16S and 23S gene sequences from single reads allowing strain level resolution of microbial communities, and improve diversity, divergence and phylogenetic estimations [117,118,119,120,121]. Depending on the primers used, sequencing of the RRN operon also enables identification and classification of Archaea and Bacteria from the same libraries [122]. However, the recent nature of developments means there are new challenges within the field, and the methods are yet to be applied to fermented foods. One such challenge is that long-read sequencing has a higher raw error rate compared to short-read sequencing. Custom-made bioinformatic pipelines are being developed specifically to reduce error rates within RRN operon sequencing [122]. With long PCR products, chimerism can also be problematic for which Unique Molecular Identifiers (UMIs) have been identified that can be useful to generate highly accurate long amplicons [123]. Additionally, the unlinked arrangement of the 16S and 23S genes in the genomes of soil bacteria presents a challenge to the scope of RRN amplicon-based community profiling in environmental samples [124]. Metabarcoding, being a highly database-dependent approach, requires large and regularly maintained databases to accurately perform taxonomical classifications [125]. Therefore, with RRN amplicon sequencing providing improved resolution and taxonomic characterisation of microbial communities, the presence of an RRN database is crucial. Taxonomic classification using RRN long reads have been performed majorly using the rrn database that searches bacterial strains based on the 16S, 23S, 5S, ITS and tRNA copy numbers, or through modified pipelines of existing 16S databases such as NCBI and SILVA to suit RRN application [120,122,126]. A commercially available RRN database named Athena along with the bioinformatic pipelines required to process and analyse long RRN amplicon reads has recently been added to the market by Shoreline in collaboration with PacBio, access to which can be obtained on purchasing their DNA extraction and library preparation kits [127]. To our knowledge, to date only one freely available reference database, named MIrROR, currently exists for RRN operon-based profiling applications [128]. The bioinformatic methods used to process and analyse long amplicon sequencing data also differ from those used for short 16S reads. OTU and ASV-based methods can be inconsistent for long reads leading to uncertainty in microbial classification and abundance calculations [129]. Presently minimap2 and BLAST, a very early aligner, are the most commonly used alignment tools to perform taxonomic assignment of long amplicon data [130,131]. While more tools are being developed, many are yet to be benchmarked preventing long amplicon sequencing from realising its full potential. Wider adoption of long amplicon sequencing will lead to its development and standardisation.

### 7.2. Untargeted or Shotgun Metagenomic Sequencing

Unlike metabarcoding methods, shotgun metagenomics approaches provide sequence data on all of the DNA content of a given sample allowing a number of genes and genome characteristics to be identified that can otherwise be complex to profile using amplicon-based methods. While tools such as PICURSt2 [132] and Tax4Fun [133] exist to functionally profile microbes using 16S sequencing data, it can be difficult to obtain strain level resolution and account for mobile genetic elements such as Horizontal Gene Transfers (HGT) using these tools [97]. Therefore, functional profiles obtained from shotgun metagenomics are superior to metabarcoding and can be useful in identifying secondary metabolites, bacteriocin gene clusters, complex metabolic pathways and interactions between pathways in microbial communities [19]. While the large amounts of sequencing data generated by shotgun metagenomics is beneficial as mentioned above, it is also more complex to process and analyse making the method computationally heavy and expensive [134]. The advantages and disadvantages of shotgun metagenomics when compared to metabarcoding are highlighted in Table 1.

Following sequencing, the raw data generated from shotgun sequencing is first passed through quality control steps. Tools such as TrimGalore, KneadData and Bowtie 2 are commonly used for adapter removal, quality trimming and host DNA removal for shotgun data generated on an Illumina platform [16,135]. Taxonomic and functional profiling can then be carried out in two ways on shotgun data, one through direct or assembly-free methods, such as Kaiju [136], Kraken [137,138] and Metaphlan [139] that assign reads using either amino acid sequence similarity, lowest common ancestor (LCA) along with k-mer matching, or clade specific markers, respectively [16,17]. Each pipeline used for assembly-free analysis has its own advantages and disadvantages with variations in results obtained based on the type of classifier and data used [21,140,141]. The pipeline chosen depends on the computation resources available, ease of use, along with the specific requirements of each pipeline [141]. Assembly-free methods work well if reference databases are constantly added to and maintained to include a diverse range of high-quality microbial genomes from across multiple sample types. However, as databases expand with a high number of metagenomic studies being conducted currently, assembly-free methods will need to be redesigned to enable their application with such large datasets [141,142].

Another method in shotgun sequencing is the assembly of reads to generate individual genomes of various microbial species/strains originating from metagenomic samples, called Metagenome Assembled Genomes (MAGs). MAGs can provide better microbiome resolution and can improve microbial characterisation and identification at species and/or strain level. MAG assembly for short reads uses overlapping reads to form contigs which are then sown together to form assemblies. While MAGs can be extremely informative about microbial populations, difficulties are still faced during the process of assembly [143]. Differing abundances of strains results in different levels (also known as depth, or coverage), of sequencing for the various genomes in a community. This variation in coverage, as well as variations in GC content are challenges to perform accurate genome assembly [144]. One method of improving MAGs generated from short reads is the process of binning, wherein similar reads are grouped together into bins before assembly. It can be carried out in two ways, supervised, where the reads are aligned against reference genomes, or unsupervised where genome characteristics such as k-mers can be used to construct assemblies which is especially useful in de novo assembly and characterisation [144]. metaSPAdes [145], Meta-IDBA [146], MetaBAT [147], CONCOCT [148], MEGAHIT [149], and MaxBin [150] are commonly employed assembly software programmes [16]. Most tools currently take GC content and coverage into account while binning. However, repetitive and mobile genetic elements continue to be problematic to MAG generation even when binning techniques are employed [151,152].

Long read sequencing helps to overcome problems associated with repetitive genome elements by producing reads that are long enough to span these sequences. High quality MAGs generated from long read metagenomic data can provide improved microbial community resolution down to the strain level and allow identification and taxonomic characterisation of rare microbial strains [153,154]. Long read shotgun metagenomic methods and bioinformatics pipelines are still being developed with frequent testing against mock communities, to reduce error rates, generate better quality MAGs, and improve the overall accuracy of the method [155,156]. The constantly improving nature of library preparation methods and the sequencing chemistries means that computational “gold standards” remain to be established. The steps involved in long read bioinformatic pipelines usually include additional error rate reduction and polishing steps besides the usual quality control and classification steps. Long read pipelines are therefore complex, using a combination of tools which are beyond the scope of this review, but more detailed information is available in the following reviews [73,74,130,157,158]. The potential of long read sequencing is expected to see extensive growth in the near future as technological developments continue.

**Table 1:** Metabarcoding vs. shotgun metagenomics advantages and disadvantages.

Factors	Amplicon Sequencing	Shotgun Sequencing	References
Cost and speed of analysis	**Advantages:** (1) Requires less sequencing per sample (2) Faster and financially feasible when many samples are to be analysed or when only taxonomic profiling is required (3) Bioinformatic analysis is relatively easier with many GUI-based software freely available, thereby reducing computational costs **Disadvantages:** Less data/information obtained on microbial communities	**Advantages:** Untargeted sequencing of metagenomic samples generates large amounts of data useful for functional profiling **Disadvantages:** Analysis methods involved can be time consuming and computationally heavy often requiring complex and expensive network infrastructures	[133,143]
Library prep	**Advantages:** (1) PCR-involving library preparation steps can increase template DNA numbers for low microbial populations, thereby improving their representation in the sequencing data generated (2) Improves microbial sequencing from host-derived samples **Disadvantages:** (1) PCR related biases apply such as differences in: (i) ease or rate of amplification (ii) variation in GC content (iii) copy number of 16S gene (iv) sequence variation between 16S copies within a bacterial genome (v) selection of targeted region (2) More susceptible to biasing microbial community representations in the presence of contaminating microbial strains such as those introduced into libraries from kit reagents used	**Advantages:** (1) PCR related biases also apply, but can be reduced using PCR-free library prep methods (2) Less susceptible to biasing microbial community representations in the presence of kitome contaminants **Disadvantages:** Host-derived samples need to be depleted for host DNA before sequencing, if not sequencing resources will be wasted on sequencing large proportions of host DNA and can lead to under/mis-representations of microbial communities	[16,72,82,118]
Microbial community profiling	**Advantages:** (1) Taxonomic classification possible for which computational processing and analysis is relatively simple and quick (2) For functional classification tools such as PICURSt2 and Tax4Fun exist that functionally assign species detected in a community through metabarcoding to predict microbial functional abilities **Disadvantages:** Functional profiles can only be predicted from amplicon data but is difficult for highly diverse and complex samples. The resulting profiles are often of low resolution and do not account for mobile genetic elements such as Horizontal Gene Transfers (HGT) and pathogenicity islands	**Advantages:** (1) The large amounts of sequencing data generated through shotgun metagenomics allows better functional profiling than metabarcoding (2) Better resolution of microbial community, even at strain level, can be obtained **Disadvantages:** (1) The extent and quality of the functional profiles obtained depend on the complexity of the sample community and the sequencing depth (2) Computational analysis is time consuming and requires complex network infrastructure to be set up and maintained which is expensive	[19,97,133,159,160]
Detection and classification of previously unidentified or uncharacterised genomes in a community	**Disadvantages:** Dependent on existing databases, making classification of new species and strains difficult	**Advantages:** Performance of de novo assembly allows characterisation of new species and strains and their addition to databases **Disadvantages:** MAG assembly for new species and strains can be very difficult for low abundance microbial populations and highly diverse microbial communities	[125,144]
Fungal or viral profiling	**Advantages:** (1) ITS-based fungal metabarcoding is relatively well characterized (2) PCR-based library prep can improve sequencing of low abundance viral microbial community members **Disadvantages:** (1) Requires different primers for fungal and viral community members and cannot be identified from a single library (2) PCR-based approaches for viral sequencing is restricted to similar or closely related viral families and can fail to detect new viral families	**Advantages:** Bacterial, fungal and viral sequences can be identified from a single library **Disadvantages:** (1) Fungal sub-populations or secondary symbionts are difficult to sequence (2) Only DNA-encoded viruses can be identified	[161,162,163,164]
Extra-chromosomal DNA profiling	**Disadvantages:** Plasmidome study is not possible	**Advantages:** Plasmidomes can be characterised along with gDNA **Disadvantages:** It is difficult to extract plasmid and genomic DNA together, and to computationally process and assemble reads. However, Hi-C approaches developed are allowing the linkage of plasmids to their carriage strains	[165,166,167]

## 8. New Technologies

Despite the advantages, barriers to long read sequencing still exist causing short read platforms to have a continued dominance of much of the metagenomics sequencing market. This has led to the rise of technologies such as synthetic long reads and Hi-C that use alternative library preparation methods and short read sequencers as alternatives to long read sequencing.

### 8.1. Synthetic Long Read (SLR) Sequencing

This method uses synthetic, artificial or virtual long reads generated from short read data. Loop Genomics, TELL Seq, and Illumina TruSeq Synthetic Long-Read are major contributors to the field of SLR sequencing. The three technologies use barcoding of short read sequences, during library prep, which can be virtually linked post sequencing to generate long reads [154,168]. Illumina’s latest SLR technology, Infinity, which is still in its developmental stage claims to generate 10 kb contiguous reads with reduced input requirements compared to long read sequencing platforms. Longas is another contributor to the SLR field, which uses MorphoSeq technology, wherein uniform random mutagenesis is performed. Tracking of these mutations allows linkage of the short reads informatically to generate long reads. SLR sequencing leverages the cost, quality, and accessibility benefits of short read sequencing while improving genome assembly and gap finishing abilities. This further contributes to the increase in the number of finished genomes added to public databases [169,170]. SLR has also found application in amplicon sequencing to improve microbial resolution [168].

### 8.2. Hi-C

Another approach to improve genome assembly is using Hi-C. This method takes advantage of linking co-located DNA during library preparation. It was originally used to improve genome assembly for larger genomes, but more recently has been applied to metagenomics [171,172,173,174]. During library preparation of metagenomic samples, DNA within the bacterial cell is cross-linked by binding to surrounding proteins, following which it is cut using restriction enzymes, and ligated. This allows DNA fragments from within the same cell to stick together [175]. After sequencing, the reads are then informatically assigned to the same cell, helping improve MAG generation, and linking of plasmid and phage DNA to specific host strains. Commercial options for kits and analysis pipelines are available, with Phase Genomics being a major contributor to the field.

## 9. Applications of Metagenomics in the Fermented Food Industry

As sequencing technologies are becoming more reliable, accessible, with higher throughputs and reduced costs, many food companies and regulatory bodies have moved away from culture-based and classical sequencing methods such as single nucleotide polymorphism (SNP) and multilocus sequence typing (MLST), and have generally adopted NGS alternatives [176]. The rapid analysis speeds further supported by real-time base calling and identification of microbial species, offered by third generation sequencing technologies such as ONT, allow food industries and regulatory bodies to make quick, informed decisions that are crucial to preventing and/or limiting foodborne outbreaks and bacteriophage invasions within the processing facilities [177,178,179]. Recently developed technologies such as “Read Until” in ONT platforms allow selective sequencing through the classification of the short prefix sequence of a DNA or RNA strand entering a nanopore into a target or non-target sequence. If classified as belonging to a set of target sequences, the entire strand is then base-called and analysed, and if not, the non-target strand is then rejected from the nanopore making it available to other strands [50,180,181]. This technology can further improve analysis speed while extending flow cell life-span and reducing sequencing costs. The “Read Until” technology has potential application in the fermented food industry specifically in screening for industrial and health-related harmful and beneficial traits.

A number of metagenomic studies have linked the presence of various genes and the metabolic pathways involved to harmful or beneficial traits possessed by microbial populations. Antibiotic resistance genes (ARGs), are examples of harmful trait-associated genes, which have has been flagged by the European Food Safety Authority (EFSA) as being linked to harms or concerns associated with foods [182,183,184,185,186,187]. Specific databases, such as CARD [188] and ResFinder [189], are available that screen for ARGs in sequencing data. Genes associated with flavor development and health promotion are examples of beneficial trait-associated genes. Various metabolites produce characteristic flavours and/or textures, the composition of which largely depends on the microbial community, the succession patterns and interactions within the community. Genes associated with acid and ethanol production, amino acid and sugar metabolism, lipid and protein lysis are often screened for when studying flavour development during the different stages of fermentations [3,13,183,190,191,192]. The identification of sugar, specifically lactose, metabolism-associated genes can further aid in determining the health promoting traits of a fermented food as the microbial breakdown of lactose to lactate during yoghurt fermentation helps alleviate problems linked with lactose consumption in lactose intolerant individuals [193]. Fermented food microbial communities are also suggested to promote health through immuno-modulation, improving gut barrier functions, preventing pathogen colonization of the gut, neutralizing microbial toxins, and producing antimicrobials such as bacteriocins [3,183,187,191,194,195]. The genes associated with these health promoting functions are commonly screened for when understanding the health benefits of consuming fermented foods. Genes associated with prebiotic functions, linked to the breakdown of complex nutrients to reduce inflammation and irritation in the gut, along with producing health promoting metabolites such as short chain fatty acids (SCFAs) have also been identified [196,197,198,199]. The health promoting abilities of fermentation microbes have been associated with survival in the gut. Genes associated with these strains include exopolysaccharide production (EPS), urease, bile salt hydrolase and mucin-binding protein synthesis [192,194,200]. The successful linking of specific trait-associated genes with certain harmful and beneficial properties in metagenomic projects is supported by the accurate collection of metadata, such as sample collection location, host health, fermentation conditions, and fermentation batches, which allows researchers to better characterise microbial communities and their associations with various sample types [201]. The applications of metagenomics are further expanded by its combination with other methods such as viability-based approaches mentioned above and with other meta-omics methods such as metatranscriptomics or metabolomics to characterise only viable microbes that are actively producing metabolites of interest [187,191,202].

NGS in combination with metagenomics allows the benefits of rapidly developing sequencing technologies to be applied to microbial population studies. Metagenomic NGS is valuable to the study of fermented foods because the microbiomes involved, either in the form of starter cultures consisting of a few selected strains, or as a large microbiome native to the raw materials used for example in spontaneous fermentations, are spatiotemporally dynamic within the food matrix. The strains are often involved in complex interactions such as cross-feeding of metabolites produced by one species to another, and/or in competitive or co-operative relationships with one other [203,204,205,206]. These interactions are often the cause of desirable organoleptic or health-promoting traits being imparted to the fermented food. Without these complex interactions the same desirable metabolites might not be produced leading to inconsistencies, as well as reduced organoleptic characteristics and microbial safety of the final fermentation end product. For this reason, entire microbiomes involved in fermentations need to be studied together and not as individual isolates, unlike in earlier single isolate WGS methods, wherein certain key pathways may not be expressed without the influence of neighbouring microbial community members and surrounding food matrix conditions [207]. The high throughput abilities and technological advances of NGS have made metagenomics feasible and affordable allowing its application in studying the influence of a variety of factors, such as geographic location and food facility conditions, on the fermentation microbiome and the effects they have on the fermentation process and the end products. Applying metagenomics in this manner contributes to stream-lining food processing pipelines, ensuring consistency and microbial safety, while protecting food and microbe-associated IP rights, preventing food fraud and unauthorized use of microbial strains. NGS has found widespread application in the food sector with rapid developments seen in the field and an extensive array of publications within the area, a few examples of which are listed in Table 2.

Metagenomics has shed light on the viromes present in fermented foods whereas culture-based methods allow the study of only singular phages causing fermentation flaws or singular foodborne viruses at a time [163]. Virome studies are scarce in fermented foods but should not be neglected. Fermented foods can contain numerous phages that can have a substantial effect on the fermentation process and can lead to low quality or failed fermentations and fermented end products. Similarly, virome studies have significant potential in improving fermented food safety through the detection of foodborne viruses [208,209]. However, the sequencing of viruses in fermented foods can be problematic due to their low abundance and smaller genome size compared to bacterial and fungal populations present in the food. This is especially true for foodborne viruses that do not multiply in food substrates [163]. Virus genomes can be DNA or RNA-encoded and only small percentages of viromes have been taxonomically assigned [210,211]. The direct sequencing of RNA, without first converting to cDNA, is a developing field with a few platforms, such as ONT and TERA-Seq, introducing native RNA sequencing [212,213]. However, library preparation involving RNA to cDNA conversion coupled with targeted amplification can improve representation of low abundance viral RNA [163].

The method and/or platform selected to sequence metagenomic samples plays an important role in determining the type and quality of sequencing data obtained. Sequencing platforms are selected based on the objective of a study, no. of samples, funding, and computational infrastructure available. When monitoring food safety in terms of screening food for pathogens, a large number of samples may be involved especially at the scale of food industries [214,215]. The rapid analysis timelines offered by HTS compared to culture-based methods promotes the application of metagenomics in food quality control enabling quick and informed decisions on product recall. The low sequencing and computational costs of targeted amplicon sequencing compared to shotgun-based approaches makes it the more cost effective choice when sequencing a large no. of samples [216,217]. The free availability of many Graphic User Interface (GUI)-based computational resources in targeted amplicon sequencing further reduces computational costs, circumvents the need for specialist intervention, and makes the analysis process more open to standardisation [112,218]. Where more in-depth microbiome studies are required, such as screening for bacteriocin genes, antimicrobial resistance genes (ARGs), and functionally characterising microbial communities for health promoting or organoleptic qualities, amplicon sequencing cannot provide sufficient information. Shotgun sequencing is required for these objectives [216,217,219]. Although shotgun sequencing is more expensive, there is a trade-off between the cost and information obtained [220,221]. The sequencing approach used to study fermented food authenticity and the influence of various factors on the fermentation microbiome can depend on the objective of the study and the amount of information required.

While metagenomics is proving to be beneficial, the technologies involved may not be presently accessible or affordable to every fermentation process. However, with the market for sequencing technologies expanding and sequencing costs reducing, along with workshops on metagenomics being organised in rural, developing areas, the scope for metagenomic applications in traditional fermented foods is steadily increasing [23].

**Table 2:** Applications of metagenomics and NGS in fermented foods.

Area	Application	References
Health promotion	Screening for health promoting bacteria Understanding the gut-brain axis Identifying prebiotics and their effect on host gut microbiota and health	[24,187,195] [222,223] [224,225,226,227]
Characterising fermentations	Organoleptic quality assessment through fermentation microbiome and volatile profiling Bacteriophage: (1) Detection and characterisation (2) Screening for phage resistant microbial strains	[6,162,228,229,230,231,232] [233,234,235] [5,236]
Food safety	Detection and prediction of foodborne pathogens and spoilage microbes Screening for bacteriocin gene clusters Checking for the presence of antibiotic resistance genes (ARGs)	[176,237,238] [239,240,241] [185,242,243]
Food fraud	Fingerprinting plant, animal and microbial components of food, determining food authenticity, and detection of contaminants and adulterants	[244,245,246,247]
Production analysis	Accessing the effect of the following factors on fermentations: (1) Raw materials and fermentation facility conditions (2) Variation between batches (3) Geographical location	[101,248,249,250,251,252] [230] [204,230,253]

## 10. Synthetic Biology

Metagenomics and metaproteomics together have improved the scientific community’s understanding of microbial species and aided in comprehending the vast varieties of metabolic functions they can perform. A large number of proteins, genes and metabolic pathways that were previously unidentified and/or unclassified are now (being) characterised. Constant development in the field of biotechnology, and more recently synthetic biology, has allowed genetic manipulations of microbial species at large-scale to produce desirable end products such as fuels, enzymes, growth hormones, insulin, and monoclonal antibodies [254]. The addition of CRISPR-cas9 methods to microbial genome editing options when compared to more traditional promoter and terminator, or plasmid-based genetic manipulations improves the robustness and scalability of synthetic biology [254,255]. The relative ease with which microbial cells can be handled, propagated and cultured, and the whole production process scaled up further contributes to microbes and/or microbial-derived products to be successfully applied to solve problems currently marring the food sector [256,257].

## 11. Food Waste Valorisation

**Figure 2:**
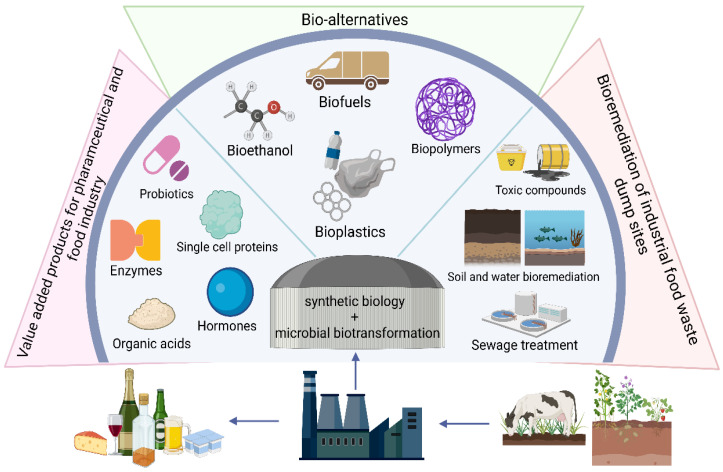
Overview of the potential value-added products that can be obtained through the combined application of metagenomics, synthetic biology and microbial biotransformation, enabling the establishment of circular bioeconomies [276,277]. In this process, metagenomics can be applied to understand the functional roles within the microbial communities to allow their application in industry through microbial biotransformation. This figure was created with BioRender.com.

A significant area of concern in the food sector is food waste. About 1.3 billion tonnes of food waste is generated along the food supply chain from farms to final consumption [258]. A substantial portion of this waste is produced by food processing facilities [259]. The waste generated is often rich in lipids, proteins and carbohydrates, the direct disposal of which can be harmful to the environment [260,261,262,263]. Many current food production methods are not sustainable and are proving to be detrimental to the environment. In order to meet the growing demand for food, current farming, agriculture and industrial food processing strategies need to be re-evaluated [264,265]. Metagenomics has the potential to help resolve these difficulties. Farm hygiene conditions, animal health and soil fertility are important factors that contribute to food safety and quality and can be linked to the microbial communities present in these environments. Metagenomics has allowed the study of these microbial communities enabling researchers to identify solutions to improving food production techniques and possibly predict and control food loses caused due to disease conditions or unnatural-disease states linked to microbial communities [266,267]. In this way metagenomics can help to prevent and reduce food waste at the farm level. The food waste streams produced by processing facilities is another point where current molecular techniques can reduce food waste [268]. Food waste streams can be used as media to culture useful microbial strains to produce value-added compounds. For this, the technologies of metagenomics, synthetic biology and microbial biotransformation can be employed. Metagenomics allows researchers to first identify microbial genes linked to the production of useful enzymes or value-added compounds [269,270,271,272]. Synthetic biology techniques would then enable the commercial application of these pathways by improving efficiency and allowing upscaling [269,270,271,272]. This way, food waste streams can be microbially-biotransformed to value-added products, paving the way for the development of circular bioeconomies (Figure 2) [273,274,275].

## 12. Future of Molecular Biology in Fermented Foods

The increased commercial interest in sequencing is leading to rapid developments within the metagenomics field. These include the development of existing and new sequencing platforms such as Element Biosciences, Singular Genomics, Omniome, Genapsys, and Ultima Genomics. These platforms can be coupled with major advancements in accompanying technologies such as library reagents, spatial profiling, single cell-technologies, and analysis pipelines. Past performance indicates that improving the efficacy and reducing the financial burden of sequencing will continue to make the technology increasingly accessible to routine applications in the food sector, leading to more widespread adoption.

## Data Availability

Not applicable.
